# Bi-anisotropic Fano resonance in three-dimensional metamaterials

**DOI:** 10.1038/s41598-018-27404-2

**Published:** 2018-06-13

**Authors:** Yuto Moritake, Takuo Tanaka

**Affiliations:** 1 Innovative Photon Manipulation Research Team, RIKEN Center for Advanced Photonics, Wako, Saitama 351-0198 Japan; 2 Metamaterials Laboratory, RIKEN Cluster for Pioneering Research, Wako, Saitama 351-0198 Japan; 3 School of Materials and Chemical Technology, Tokyo Institute of Technology, Ookayama, Tokyo 152-8550 Japan

## Abstract

We experimentally investigated the bi-anisotropic properties of Fano resonance in three-dimensional (3D) metamaterials. Fano resonance in 3D metamaterials arises from the interference of in-phase and anti-phase modes that originate from mode hybridization in coupled 3D split ring resonators (SRRs) with detuned resonant wavelengths. At Fano resonance, not only permittivity and permeability but also the bi-anisotropic parameter show doubly dispersive response. Manipulation of the bi-anisotropic response at Fano resonance was demonstrated through controlling the inversion symmetry of the 3D-SRRs. Improvement of inversion symmetry due to rotation of 3D-SRRs results in enhancement of magnetic response and inhibition of electric and bi-anisotropy responses at Fano resonance. Negligible electric and bi-anisotropic responses at Fano resonance were achieved due to the small radiative nature of the anti-phase mode. This bi-anisotropic Fano metamaterials with rich and tunable bi-anisotropy will extend the capabilities of new optical phenomena and broaden the applications of bi-anisotropic metamaterials.

## Introduction

The emergence of metamaterials has opened exciting opportunities to realize artificial materials with designed optical parameters. In optics, although only electric permittivity has been mainly discussed so far, artificial magnetism in metamaterials allows us to manipulate magnetic permeability^1–5^. Furthermore, the cross term, which is the origin of bi-anisotropy, is an additional degree of freedom to engineer the optical response in artificial materials^6^. The relationship between electromagnetic fields in bi-anisotropic materials can be described as

1 $$(\begin{array}{c}D\\ B\end{array})=(\begin{array}{cc}{\varepsilon }_{0}\varepsilon  & -i\xi /{c}_{0}\\ i\xi /{c}_{0} & {\mu }_{0}\mu \end{array})(\begin{array}{c}E\\ H\end{array})$$

where *ε*_0_, *μ*_0_, *ε*, and *μ* are vacuum permittivity, vacuum permeability, relative permittivity, and relative permeability, respectively. *c*_0_ is the speed of light in a vacuum, and *ξ* is a bi-anisotropy parameter. Eq. (1) indicates that magnetic (electric) dipoles can be excited not only by the magnetic (electric) field but also by the electric (magnetic) field. Various kinds of new and interesting optical phenomena have been observed due to bi-anisotropy such as strong optical activity, topological electromagnetic states in photonics, and so on^7–9^.

From a structural point of view, bi-anisotropic response in metamaterials originates from the structures’ lack of inversion symmetry along the direction of light propagation (generally assigned to the *z*-axis)^10,11^. Therefore, planar structures, which are frequently used in metamaterial studies, have, in principle, no bi-anisotropy although there is a small amount of bi-anisotropy due to the presence of the substrate that breaks the inversion symmetry. On the other hand, three-dimensional (3D) structures have a great potential to achieve and control the bi-anisotropic response in metamaterials^10–18^. So far, we have demonstrated that 3D split ring resonators (SRRs) show bi-anisotropic response at magnetic resonance and controllability of this bi-anisotropic response through inversion symmetry^17,18^. By rotating single-cut 3D-SRRs, inversion symmetry along the light propagation direction is improved and results in purely magnetic resonance in metamaterials^18^.

**Figure 1:**
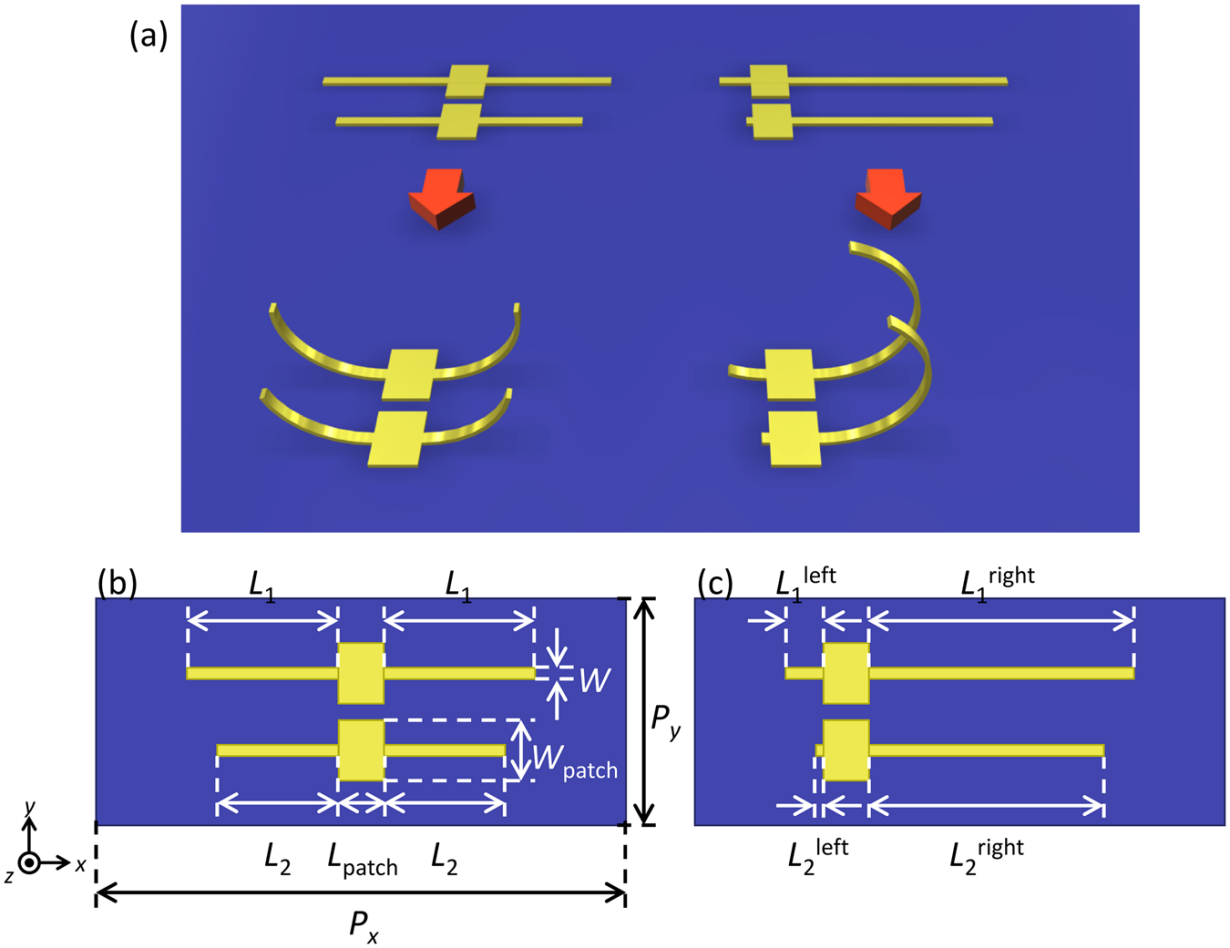
Schematics of coupled 3D-SRRs. (**a**) Planar gold patterns and self-folded 3D-SRRs on a silicon substrate without (left) and with rotation (right). The design of the planar gold patterns for 3D-SRRs (**b**) without and (**c**) with rotation.

To extend the capabilities of bi-anisotropic metamaterials, one important question is how bi-anisotropy appears at a more complex resonance such as Fano resonance^19–23^. The purpose of this study is to show the bi-anisotropic properties and its controllability of Fano resonance in 3D metamaterials. Wu *et al*.^15^ have reported mode hybridization in coupled 3D-SRRs with detuned resonant wavelengths fabricated by a multiple lithography process. Here, we also investigated coupled 3D-SRRs shown in Fig. 1(a) fabricating them by a metal-stress-driven self-folding method. The self-folding method allows the formation of circular 3D-SRRs and easy control of the 3D-SRRs’ orientation via rotation, which is advantageous for manipulating bi-anisotropy. The resonant wavelength detuning of 3D-SRRs is introduced by the length difference of the arms, giving rise to mode hybridization of magnetic modes and Fano interference effect. Effective parameter retrieving shows bi-anisotropic response at Fano resonance. Moreover, by improving the inversion symmetry of the 3D-SRRs, controlling electric, magnetic, and bi-anisotropic response at Fano resonance is demonstrated.

## Results and Discussion

For experimental realization of the 3D-SRRs, metal-stress-driven self-folding method^17,18,24,25^ as shown in Fig. 1(a) was employed in this study. By applying selective and isotropic etching to a silicon substrate with planar gold patterns, the gold patterns are released from the substrate and bend spontaneously by residual intrinsic stress of the deposited gold film. Intrinsic stress in the gold film is due to various reasons including lattice mismatch, grain boundaries, difference of thermal expansion coefficient, impurities in the gold film, and deposition method^19^. Since thermal evaporation without substrate heating is utilized in our fabrication, the temperature of the gold and the substrate is different during deposition, resulting in a shrinking force in the metal film after the sample was cooled to room temperature. Advantages of the self-folding method are (i) mass and easy fabrication of 3D structures, (ii) circular shape of fabricated 3D-SRRs, and (iii) easy manipulation of the orientation via rotation of 3D structure by shifting the patch from the center as shown in Fig. 1(a). Details on the conditions of lift-off and etching are explained in the Fabrication subsection of the Method section found at the end of this article.

The planar gold patterns before etching are shown in Fig. 1(b). A single pattern is composed of arms and a center patch. The arms are the folding parts and the center patch connecting the left and right arms works as an anchor so that the structure would not be peeled off from the substrate after etching. The considered metamaterials are composed of pairs of 3D-SRRs with different arm lengths, Δ*L* = *L*_1_ − *L*_2_ (Fig. 1(b)), where *L*_1_ is the arm length of the first 3D-SRRs and *L*_2_ is the length of the second 3D-SRRs. Each pair of 3D-SRRs is considered as a coupled system. The length difference Δ*L* is varied from 0 to 0.6 μm to investigate the dependence of the coupled 3D-SRRs’ optical properties on the degree of structural asymmetry. The design of planar gold patterns is summarized in Table 1. The arm length difference detunes the resonant wavelengths of 3D-SRRs and makes the coupled systems asymmetrical, giving rise to excitation of optically dark modes formed by mode hybridization. Interference between optically bright and dark modes results in Fano resonance.

**Table 1:** The structural parameters of gold patterns for 3D-SRRs without rotation. (Unit: μm).

Δ*L*	*L*_1_	*L*_2_	*w*	*L*_patch_	*W*_patch_	*P*_*x*_	*P*_*y*_
0.0	2.0	2.0	0.15	0.6	0.8	7.0	3.0
0.2		1.8					
0.4		1.6					
0.6		1.4					

To demonstrate controllability of the bi-anisotropic response, coupled 3D-SRRs with rotation are also considered. Here, rotation refers to the rotation of the 3D-SRRs with respect to the the *y*-axis ((Fig. 1(a)). To rotate the 3D-SRRs, the center patch is shifted along the arms by 1.5 μm as shown in Fig. 1(c), resulting in rotation of the 3D-SRRs after self-folding as shown in Fig. 1(a). The gap between the arms of the rotated 3D-SRRs approaches the side of the 3D-SRRs inducing inversion symmetry along the *z*-axis. The design of the planar gold patterns for the rotated 3D-SRRs is summarized in Table 2. Because of the structural restriction for the rotated structures, the length difference of *L*_1_^left^ for Δ*L* = 0.6 μm is set to 0.5 μm.

**Table 2:** The structural parameters of gold patterns for 3D-SRRs with rotation. (Unit: μm).

Δ*L*	*L*_1_^left^	*L*_2_^right^	*L*_1_^left^	*L*_2_^right^	*w*	*L*_patch_	*W*_patch_	*P*_*x*_	*P*_*y*_
0.0	0.5	3.5	0.5	3.5	0.15	0.6	0.8	7.0	3.0
0.2			0.3	3.3					
0.4			0.1	3.1					
0.6			0.0	2.9					

**Figure 2:**
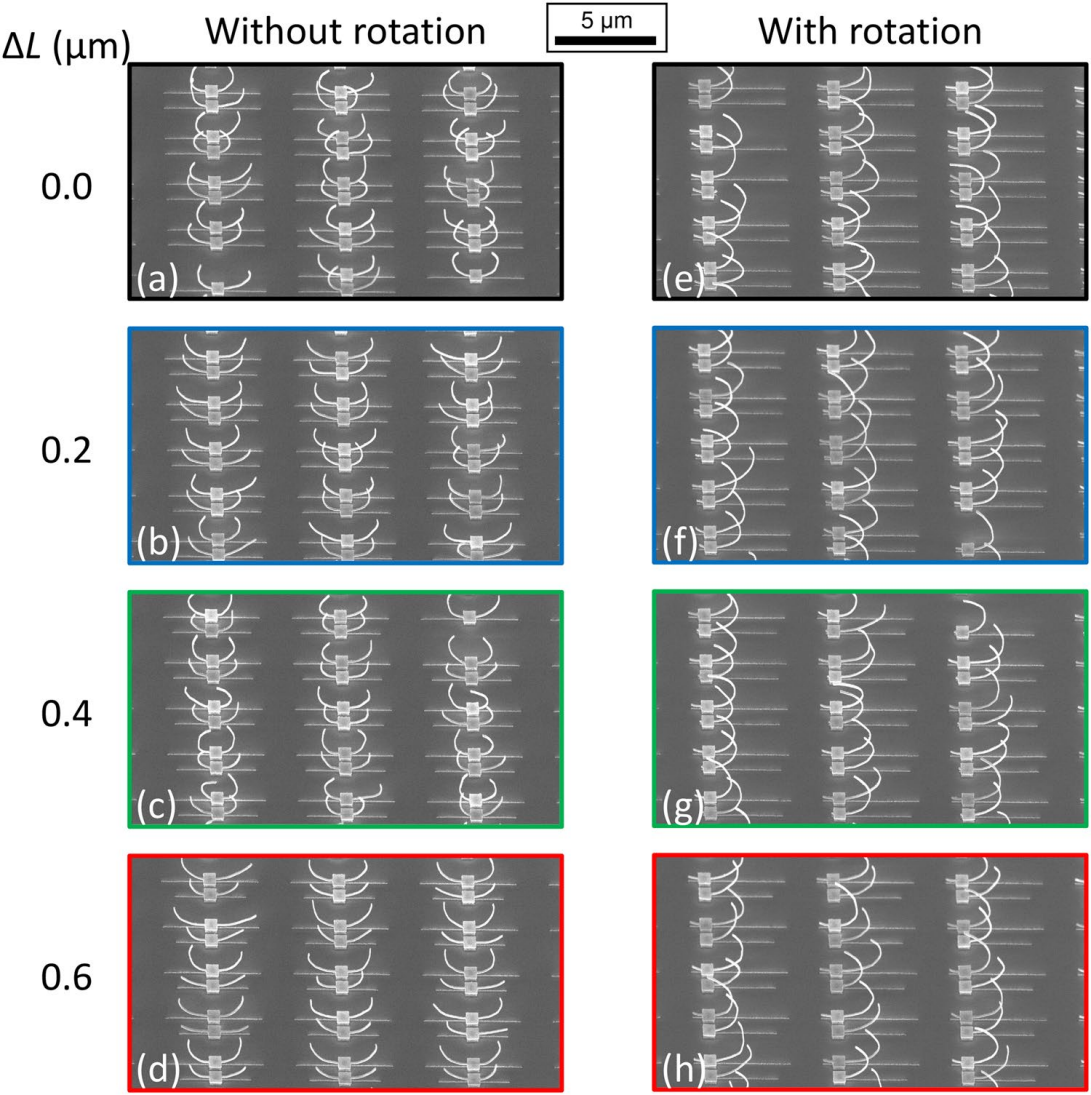
SEM images of the fabricated metamaterials composed of coupled 3D-SRRs (**a**–**d**) without and (**e**–**h**) with rotation. Δ*L* (in μm) for (**a**,**e**) = 0.0, for (**b**,**f**) = 0.2, for (**c**,**g**) = 0.4, and for (**d**,**h**) = 0.6. Colored borders of each figure refers to the color code used in Fig. 3.

**Figure 3:**
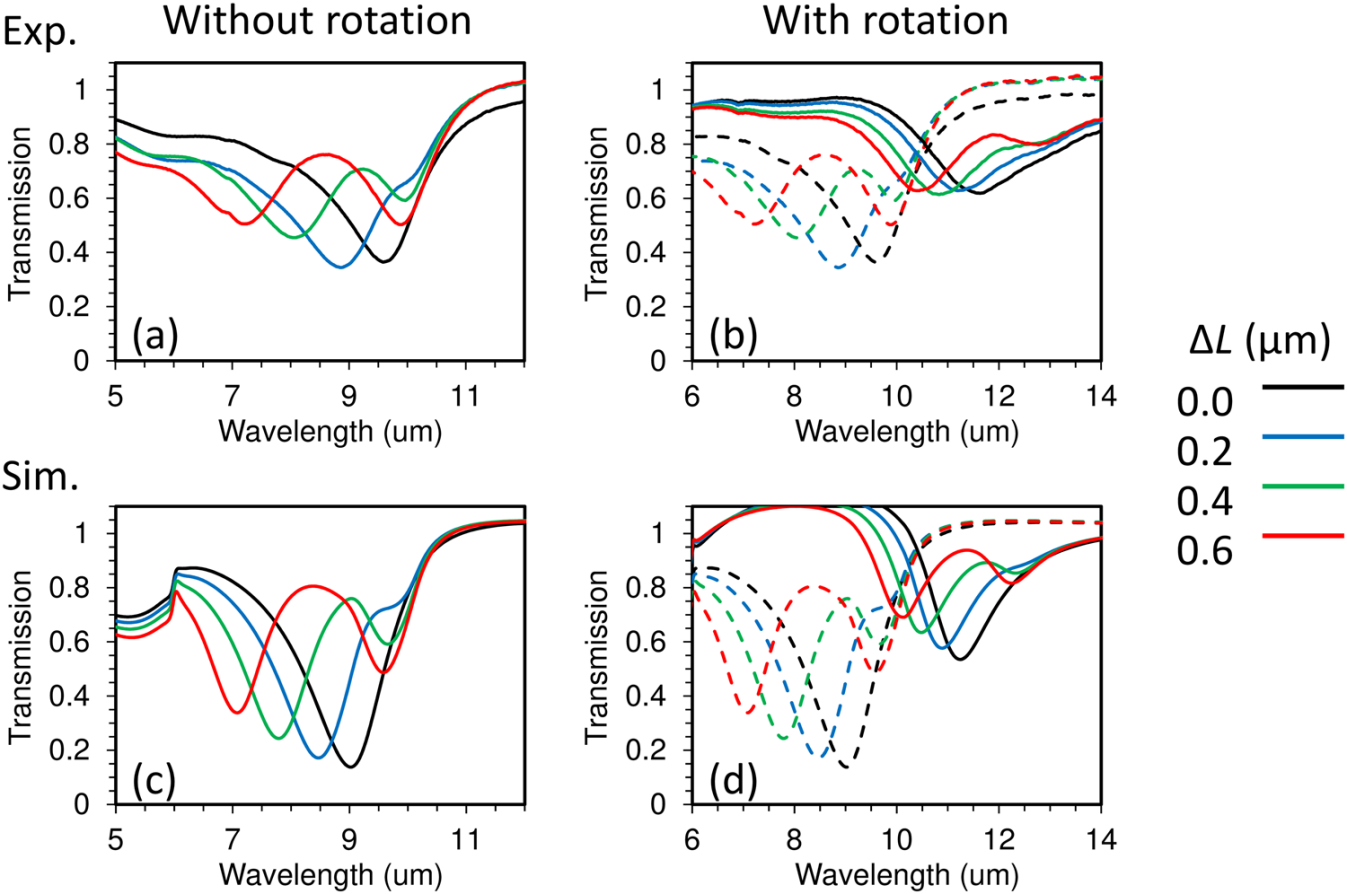
Transmission spectra for metamaterials composed of coupled 3D-SRRs (**a**,**b**) experiments and (**c**,**d**) simulation. Experimental results are measured by using FTIR spectrosvopy and simulated results was obtained by using COMSOL. (**a**,**c**) without rotation and (**b**,**d**) with rotation. The black, blue, green, and red solid lines correspond to Δ*L* = 0.0, 0.2, 0.4, 0.6 μm, respectively. The dashed lines in (**b**,**d**) indicate the results in the case of without rotation.

Figures 2 and 3 show scanning electron microscope (SEM) images of fabricated metamaterials and measured transmission spectra, respectively. Fourier-transform infrared (FTIR) spectroscopic measurements was used for optical characterization. Illuminated light was incident normally to the substrate and direction of the polarization was along *x-*axis (parallel to the gap and arms of the 3D-SRRs). Illuminated area is 40 × 40 μm (about 6 × 13 unit cells are illuminated). All transmission spectra are normalized by that of a bare silicon substrate with around 70% transmission to eliminate the absorptive effect of the substrate. This is why some transmission values exceed 100%.

First of all, we discuss the optical response in the coupled 3D-SRRs without rotation. When Δ*L* = 0.0 μm (black line in Fig. 3(a)), only one resonance corresponding to the magnetic mode excitation is observed at around 9.5 μm. Increasing Δ*L*, Fano resonance appears at around 10 μm. The Fano resonance is a result of the interference between in-phase and anti-phase magnetic modes originating from mode hybridization as discussed later. Next, we move to the optical response of the 3D-SRRs with rotation. While resonances become red shifted and shallow, the main features of the spectra are similar compared with the 3D-SRRs without rotation. The red shift is because one end of the ring, where electric fields concentrate at resonance, approaches the silicon substrate, which has a high refractive index of 3.4. Shallowing of the transmission dips (high transmission at resonance) is due to improvement of impedance matching through reduction of resonance in electric permittivity as shown in the later part of this article.

**Figure 4:**
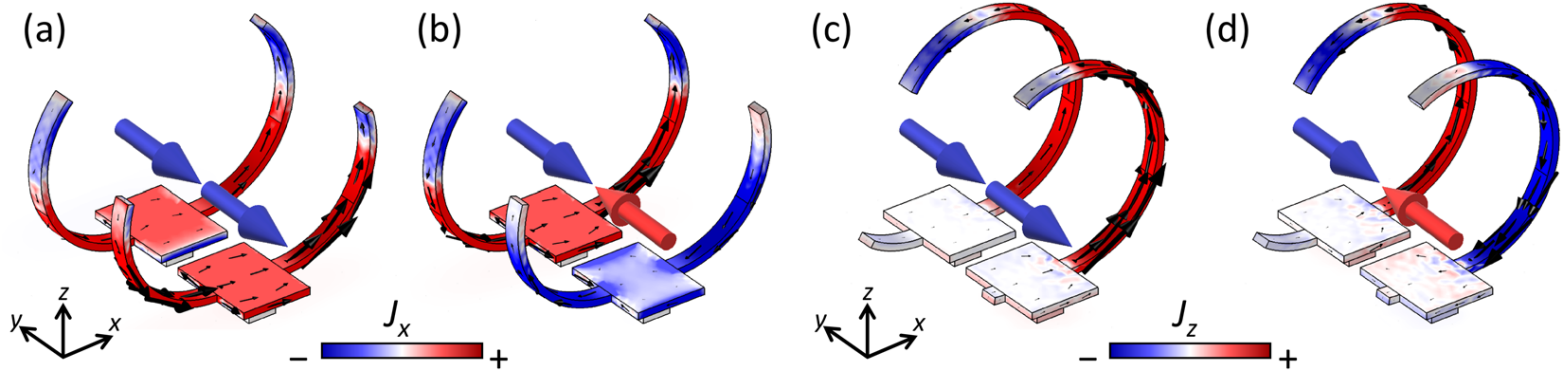
The simulated current distributions at resonance in the case of Δ*L* = 0.4 μm. (**a**) In-phase (7.72 μm) and (**b**) anti-phase (9.68 μm) modes in the 3D-SRRs without rotation. (**c**) In-phase (10.60 μm) and (**b**) anti-phase (12.17 μm) for the 3D-SRRs with rotation. Black arrows indicate direction and amplitude of induced currents. Color maps show (**a**,**b**) *x*- and (**c**,**d**) *z*- components of induced currents. Blue and red arrows indicate the direction of induced magnetic dipoles.

To investigate the electromagnetic modes in the coupled 3D-SRRs, simulations were carried out by using commercial software based on a finite-element method (COMSOL). For more details on the simulations, see the Simulation subsection of the Method section found at the end of this article. The simulated transmission spectra show good agreement with experiments (Fig. 3(c,d)). The fabricated 3D structures are not uniform as shown in Fig. 2, resulting in inhomogeneous broadening of transmission dips in experiments (Fig. 3). Although deformation of the arms affects the resonant wavelength, the resonant wavelength is mainly determined by the length of the arm in our structures. Therefore, the resonance is robust against structural deformation. Figure 4 shows the induced current distributions for the case of Δ*L* = 0.4 μm at resonance. Figure 4(a,b) correspond to current distributions in the 3D-SRRs without rotation at in-phase (7.72 μm) and anti-phase (9.68 μm) modes, respectively. Black arrows indicate directions and amplitudes, and color plots indicate *x*-components. At the in-phase modes, induced currents oscillate in phase, while anti-phase currents flow at the anti-phase mode. Since these currents induce magnetic dipoles along the *y*-axis at each ring (blue and red arrows in Fig. 4), a magnetic quadrupole is formed at the anti-phase mode and works as an optically dark mode in Fano resonance. Although the anti-phase mode is formed in the case of Δ*L* = 0.0 μm, perfect symmetry of the structures makes the mode completely dark, resulting in no signal of the resonance in the spectra. Figure 4(c,d) show current distributions in the 3D-SRRs with rotation at in-phase (10.60 μm) and anti-phase (12.17 μm) modes, respectively. Black arrows indicate directions and amplitudes, and color plots indicate *z*-components. A similar behavior to that of the 3D-SRRs without rotation is observed, therefore, the rotation does not break Fano resonance in coupled 3D-SRRs.

In order to study the bi-anisotropic properties of 3D-SRRs, effective permittivity, permeability, and bi-anisotropic parameters of the metamaterials were retrieved by following ref.^22^. The effective parameters were retrieved using complex transmission and reflection coefficients calculated by COMSOL. For details of the parameters retrieving, see the Effective Parameter Retrieving subsection in the Method section found at the end of this article.

**Figure 5:**
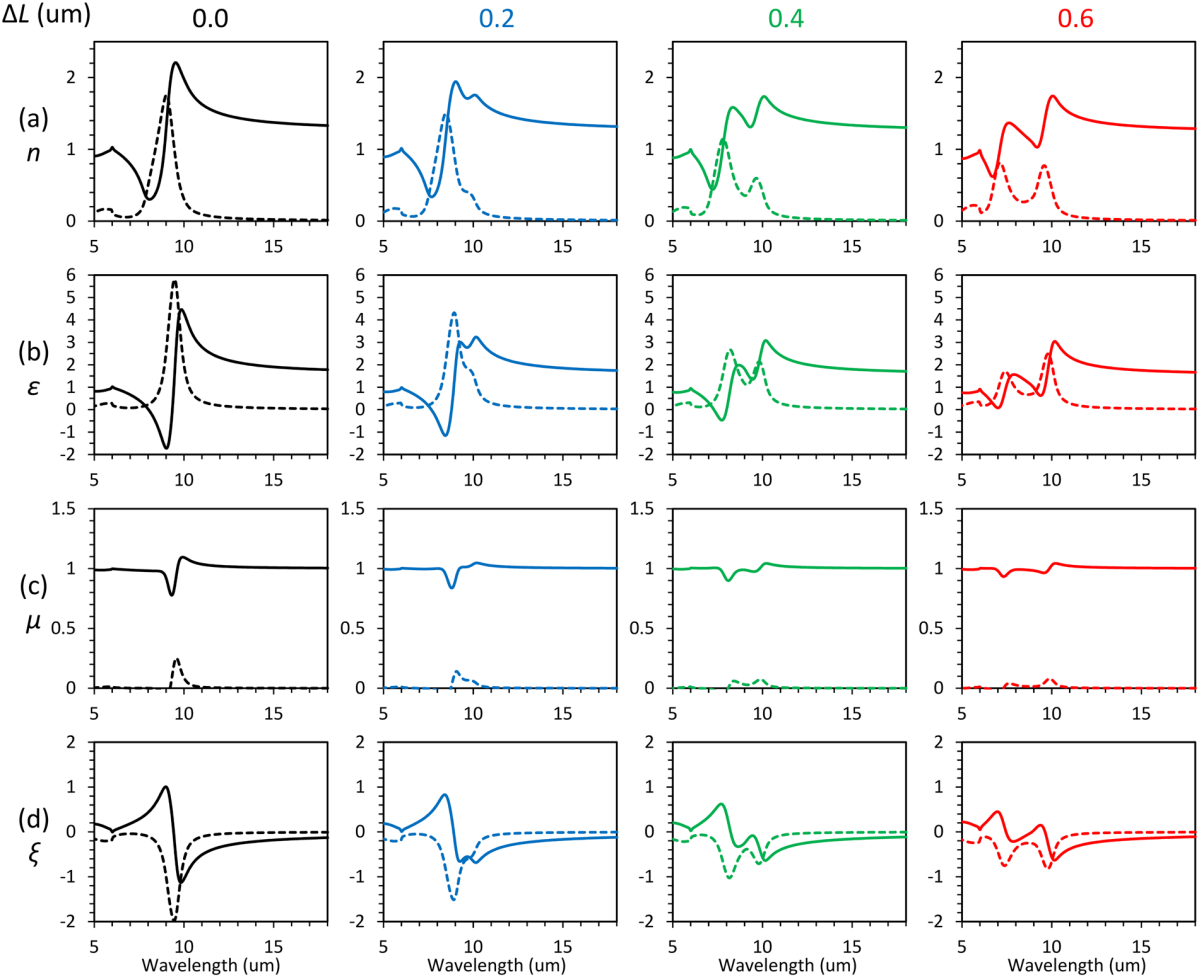
Δ*L* dependence of retrieved effective parameters extracted from simulation in the case of 3D-SRRs without rotation. (**a**) Refractive index (*n*), (**b**) relative permittivity (*ε*), (**c**) relative permeability (*μ*), and (**d**) bi-anisotropic parameter (*ξ*). Solid and dashed lines indicate real and imaginary parts of effective parameters, respectively.

The retrieved effective parameters for the 3D-SRRs without rotation are summarized in Fig. 5. For Δ*L* = 0.0 μm, only single resonance due to the in-phase mode is observed. Large magnitude of resonance in *ε* and small magnitude of resonance in *μ* insist the resonance is almost electric rather than magnetic. Since the structures have no inversion symmetry along the propagation direction (*z*-axis), *ξ* shows non-zero values at resonance. Increasing Δ*L*, we can observe a doubly dispersive response originating from Fano resonance in all parameters. The doubly dispersive response in *ξ* indicates that cross coupling between electric and magnetic fields exists at Fano resonance, which is the reason why we call the resonance as bi-anisotropic Fano resonance. Resonant amplitudes for all optical parameters are large when Δ*L* = 0.0 μm because two SRRs have the same resonant frequency and spectrally overlap each other. When Δ*L* increases, the resonant frequency of the two SRRs are detuned and the resonant amplitude is reduced.

**Figure 6:**
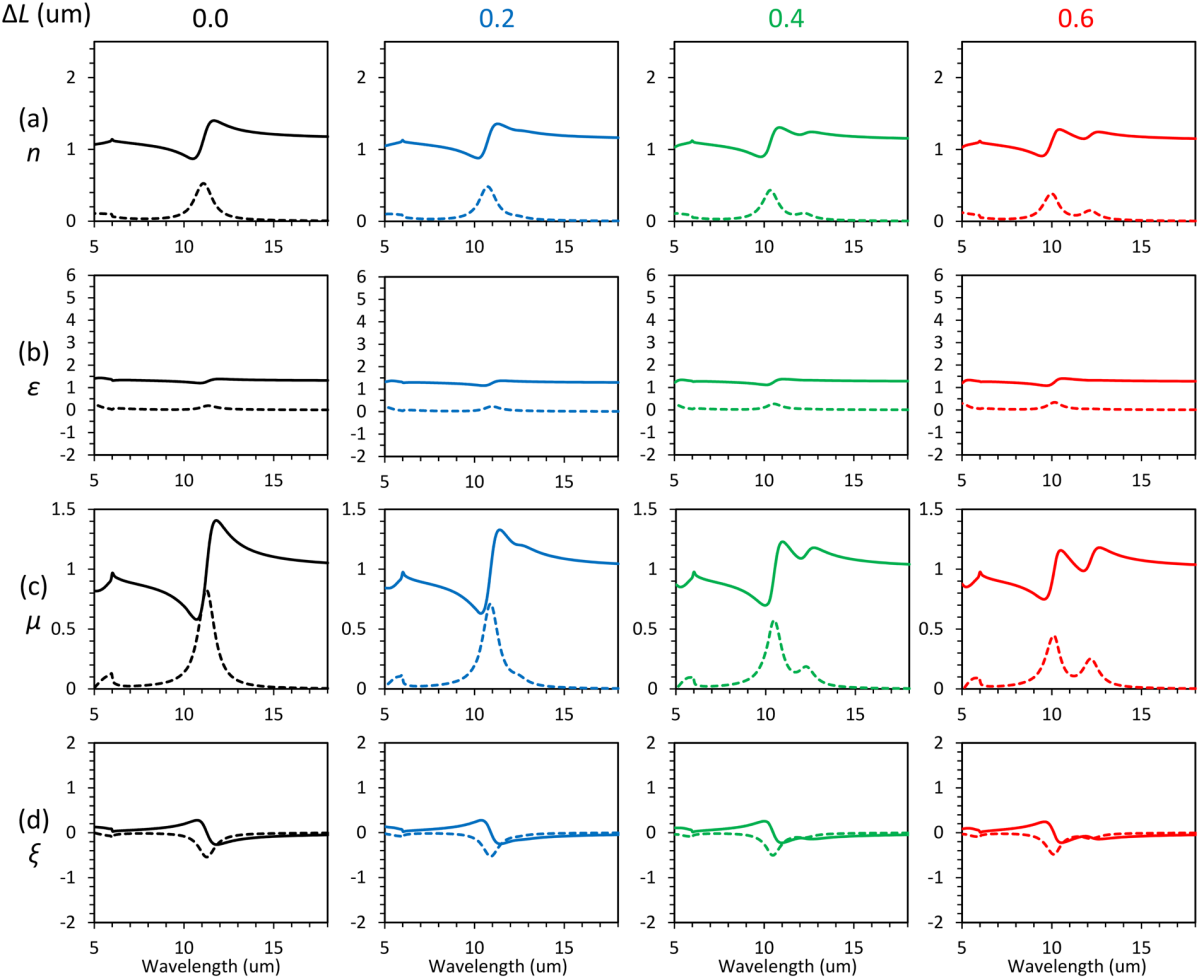
Δ*L* dependence of retrieved effective parameters extracted from simulation in the case of 3D-SRRs with rotation. (**a**) Refractive index (*n*), (**b**) relative permittivity (*ε*), (**c**) relative permeability (*μ*), and (**d**) bi-anisotropic parameter (*ξ*) for different Δ*L*. Solid and dashed linesindicate real and imaginary parts of effective parameters, respectively.

Lastly, we investigate the change in effective parameters by rotating the structures to show controllability of the electromagnetic response in bi-anisotropic Fano resonance. Figure 6 shows the retrieved effective parameters for the 3D-SRRs with rotation. The most drastic change from Fig. 5 is the dramatic enhancement of *μ* and inhibition of *ε* and *ξ* due to improvement of inversion symmetry, which means the resonance approaches magnetic resonance from electric resonance^18^. Since the induced electric dipoles are parallel to the gap direction of 3D-SRRs, rotation of the structures makes electric dipoles normal to the surface of the substrate. Therefore, an electric component of incident light cannot couple with the mode through the electric dipole excitation, and it eliminates alteration in *ε* and *ξ*. Since the presence of *ξ* negatively affects amplitude of resonance in *μ*^2,26^, enhancement of *μ* is observed by improving inversion symmetry. At the anti-phase mode, the resonant amplitude of *ε* and *ξ* are negligibly small although dispersive shapes are still observable at the in-phase mode (Fig. 6(b,d)). This is because the cancelation of electric dipoles at the anti-phase mode makes coupling through an electric component of light smaller in addition to the reduction due to improved inversion symmetry.

## Conclusions

We experimentally investigated the bi-anisotropic properties of Fano resonance in metamaterials composed of coupled 3D-SRRs. Fano resonance in 3D metamaterials arise from the interference of in-phase and anti-phase modes that originate from mode hybridization in coupled 3D-SRRs with detuned resonant wavelengths. 3D-SRRs with different Δ*L* were fabricated by using a self-folding method to investigate dependence of the coupled 3D-SRRs’ optical properties on its degree of asymmetry, and showed Fano resonance in the near infrared region. Effective parameter retrieving for bi-anisotropic materials revealed that not only permittivity and permeability but also the bi-anisotropic parameter showed doubly dispersive response due to Fano resonance. Moreover, manipulation of bi-anisotropy at Fano resonance was demonstrated by controlling inversion symmetry of the 3D-SRRs. Owing to the self-folding method, the orientation of 3D-SRRs can be easily controlled through changing the patch position along the arms. Improvement of inversion symmetry resulted in enhancement of magnetic response and inhibition of electric and bi-anisotropic response at Fano resonance, which indicated controllability of the bi-anisotropic properties in 3D Fano metamaterials. Negligible electric and bi-anisotropic responses at Fano resonance was achieved due to the small radiative nature of the anti-phase mode. Bi-anisotropic Fano metamaterials with rich and tunable bi-anisotropy demonstrated in this study will extend the capabilities of new optical phenomena and broaden the applications of bi-anisotropic metamaterials.

## Method

### Fabrication

First, planar gold patterns were formed on a Si substrate by using a conventional lift-off method. Patterning on a resist and evaporation of 60 nm-thick gold were performed via electron beam lithography and thermal evaporation, respectively. Prior to gold deposition, buffered hydro fluid treatment was performed to get better adhesion between silicon and gold. After forming the structures on the silicon substrate, inductive coupled plasma reactive ion etching (ICP-RIE, SAMCO, RIE200iPT) was performed with the following parameters: CF_4_ plasma of flow rate of 20 sccm, pressure of 0.15 Pa, ICP power of 500 W, and forward power of 0 W. By setting the forward power to 0 W, only the silicon substrate was isotropically etched.

### Simulation

All simulations were performed using commercial software based on a finite element method (COMSOL). We used the dielectric function of gold from ref.^27^ and set the refractive index of silicon to 3.4. The structural parameters were set to be the values in Tables 1 and 2. The curvature of the arms is 0.85 μm, measured from the fabricated structures. The 3D-SRRs are on a square pillar structure to take into account etching of a silicon substrate. The lengths of the silicon pillars along *x*, *y*, and *z*-axis are 0.24, 0.44, and 0.18 μm, respectively. All simulated transmission spectra were normalized by that of the bare silicon substrate with 70% transmission for direct comparison with experiments.

### Effective parameter retrieving

Effective parameter retrieving were carried out using the S-parameters that were automatically calculated by COMSOL. The S-parameters were calculated for both incident directions (from air to substrate and from substrate to air) to get four complex coefficients, *t*^+^, *r*^+^, *t*^−^, and *r*^−^. These parameters were substituted into the analytic equations in ref.^6^.
